# Addressing Discrimination and Violence against Lesbian, Gay, Bisexual, Transgender, and Queer (LGBTQ) persons from Brazil: A mobile Health intervention

**DOI:** 10.21203/rs.3.rs-2034975/v1

**Published:** 2023-03-28

**Authors:** Monica Malta, Angelica Baptista Silva, Cosme Marcelo Furtado Silva, Sara LeGrand, Michele Seixas, Bruna Benevides, Clarisse Kalume, Kathryn Whetten

**Affiliations:** Centre for Addiction and Mental Health; National School of Public Health, Oswaldo Cruz Foundation; National School of Public Health, Oswaldo Cruz Foundation; Duke University; UN Women Brazil; National Association of Travestis and Transsexuals (ANTRA); Micro Rainbow International Foundation; Duke University

**Keywords:** mobile health, mHealth, mobile app, LGBT, LGBTQ+, gender-based violence, discrimination, IPV

## Abstract

**Background:**

Sexual and gender minorities (SGM) experience higher rates of discrimination and violence when compared to cis, heterosexual peers. However, violent crimes and other hate incidents against SGM persons are consistently not reported and prosecuted because of chronic distrust between the SGM community and police. Brazil is one of the most dangerous countries for SGM persons in the world. Herein we describe the development of a mobile health intervention to address the rampant violence against this population, the Rainbow Resistance - Dandarah app.

**Methods:**

We conducted community-based participatory research (CBPR) between 2019–2020. The study started with in-depth interviews (IDIs) and focus group discussions (FGDs) with representatives of the SGM community from Brazil. Descriptive qualitative data analysis included the plotting of a ‘word cloud’, to visually represent word frequency, data coding and analysis of more frequent themes related to app acceptability, usability, and feasibility. A sub-sample of SGM tested the app and suggested improvements, and the final version was launched in December 2019.

**Results:**

Since the app was launched in December 2019, the app recorded 4,114 active SGM users. Most participants are cisgender men (50.9%), self-identified as gay (43.5%), White (47.3%), and aged 29 or less (60.9%). FGDs and IDIs participants discussed the importance of the app in the context of widespread violence toward SGM persons. Study participants perceived this mHealth strategy as an important, effective, and accessible strategy for SGM surviving violence. The CBPR design was highlighted as a key strategy that allowed SGM persons to collaborate in the design of this intervention actively. Some users reported how the panic button saved their lives during violent attacks.

**Conclusions:**

Rainbow Resistance - Dandarah app was endorsed as a powerful tool for enhancing reporting episodes of violence/discrimination against SGM persons and a key strategy to connect users with a safe network of supportive services. Results indicate that the app is an engaging, acceptable, and potentially effective mHealth intervention. Participants reported many advantages of using it, such as being able to report harassment and violence, connect with a safe network and receive immediate support.

## Introduction

Over the past decade, Latin America has stood out for recognizing sexual and gender minority (SGM) rights. Outside of the Caribbean, most countries in the region have decriminalized same-sex sexual acts between consenting adults. In most countries, SGM individuals are constitutionally protected from discrimination based on their gender and/or sexual orientation. Latin America has also made impressive progress on marriage equality. In 2010, Argentina became the first country in the region to approve same-sex marriage, followed by Brazil and Uruguay in 2013, Colombia in 2016, Ecuador in 2019, Costa Rica in 2020, and Chile in 2021 (Malta et al., 2019).

However, several obstacles stand in the way of further progress. SGM activists face backlash from social and religious conservatives, and some leaders, including Presidents Nayib Bukele of El Salvador and former Brazilian president, Jair Bolsonaro, are openly hostile to LGBTQIA+ rights. With hundreds of unprosecuted hate crimes, endemic violence and discrimination, Latin America continues to lag in SGM rights among rampant violence against the community. The average life expectancy of trans women in Latin America is only 35 years, according to a report by the Inter-American Commission on Human Rights - compared to 80 years among the general population (IACHR/OAS, 2018).

The Trans Murder Monitoring project registered a total of 4369 murders of trans and gender-diverse people in 77 countries and territories between 1 January 2008 and 30 September 2022. 68% of all the murders registered happened in Central and South America, and 29% in Brazil. (TGEU, 2022). Brazil is where more trans people are killed in the world, with at least 135 trans women killed in 2021 only, according to the Brazilian Transgender Association (ANTRA, 2022). The record-breaking figures of 2022 build on a yearly growing trend.

Experiences of discrimination, bullying and violence against SGM persons are widespread in schools, workplaces, healthcare settings, restaurants, streets, etc. (Kosciw & Zongrone, 2019). SGM persons are often excluded from legal protections, rejected by families and communities, and lack equal treatment. However, violent crimes and other hate incidents against SGM persons are consistently not reported and prosecuted because of chronic distrust between the SGM community and police (Mallory et al., 2015). This is caused by frequent episodes of discrimination and harassment by law enforcement based on sexual orientation and gender identity, an ongoing and pervasive problem (Mallory et al., 2015).

Herein we describe a mobile health intervention developed to address the rampant violence against SGM persons from Brazil and structural problems that influence underreporting of crimes.

## Methods

To develop the Rainbow Resistance - Dandarah app and address the specific needs of SGM persons from Brazil, a community-based participatory research (CBPR) was conducted between 2019–2020. The study started with in-depth interviews (IDIs) and focus group discussions (FGDs) with representatives of the SGM community from Brazil. Our major aim was to identify feasible strategies to increase the reporting of discrimination and violence episodes against SGM persons from Brazil and to connect those in need with a broad range of supportive services.

CBPR involved collective, reflective, and systematic inquiry in which researchers and SGM community members worked together in all research process steps. CBPR is a key methodology for bringing about social change and is particularly useful when working with highly marginalized populations such as SGM in Brazil (Israel et al., 1998; Baum et al., 2006; Tremblay et al., 2018). Twenty-two FGDs were conducted with SGM persons from seven states in Brazil (Pará, Minas Gerais, Sergipe, Rio de Janeiro, São Paulo, Distrito Federal and Bahia) and eight cities: Belém, Aracaju, Rio de Janeiro, Niterói, Uberlândia, Brasília, Salvador, and São Paulo, including 300 SGM. Then IDIs were also conducted with a few key informants, most community leaders and professionals providing services for SGM persons experiencing discrimination/violence.

Following this first phase, the app was developed and pilot tested by ten members of the SGM community in Brazil. Those participants pilot-tested the app to determine users’ comprehension of the educational content, understanding and use of intervention features, and overall impressions of app relevance and appeal. Participants were asked to share their thoughts and impressions as they moved through the different components and features of the app. After this pilot test, a new review and features tailoring round were conducted before the app was launched in December 2019. The final app included the following features: violence registration, report, mapping, a panic button, and features offering 24/7 support to violence victims.

Community Advisory Board: Eleven participants from the Brazilian SGM community participated in the study Community Advisory Board (CAB). Members of the CAB represented the broad diversity of the SGM community. They met once a month, collaborating with the research team to develop culturally sensitive and adequate recruitment strategies, adapt study protocols and tools, and contribute to interpreting and disseminating research findings.

Analysis of IDIs and FGDs: The IDIs and FGDs were audio-recorded and transcribed. Descriptive analysis included the plotting of a world cloud with more frequent words utilizing an extension of the statistical software R version 4.1.1 (rquery.wordcloud). All transcripts were reviewed and independently analyzed by two investigators (MM & ABS), and themes/coding discrepancies were discussed to reach an agreement. During this process, the domain structure was continually reassessed and underwent subsequent revisions. The app features were based on results of this qualitative analysis and major themes identified. The Rainbow Resistance - Dandarah App was then developed to allow users to register, denounce? and map acts of violence, discrimination and hate crimes towards SGM persons from Brazil.

Results from IDIs and FGDs were categorized into the following domains: acceptability, anticipated usability, and feasibility. Acceptability was defined as the willingness of SGM persons to use the Rainbow Resistance- Dandarah app. Anticipated usability was defined as the extent to which SGM persons found the program useful to report violent episodes and access support. Feasibility was defined as the extent to which the program could be made readily available and implemented among the SGM from Brazil. In addition, feedback on the app’s look, feel, and content was also gathered to enhance acceptability.

This was a pilot study, and our major goal was to establish Rainbow Resistance - Dandarah app acceptability and feasibility, utilizing a mixed-methods strategy that compared and combined qualitative (FGDs, IDIs) and qualitative results (e.g., socio-demographic characteristics, panic button usage etc.). Below we describe the major results of the study.

## Results

### Socio-demographic characteristics of study participants

Since the app launch, in December 2019, until August 2022, 4,114 active SGM users have been recorded. Most participants are cisgender men (50.9%), self-identified as gay (43.5%), White (47.3%), and aged 29 or less (60.9%) (Table 1).

### Descriptive results from Focus Groups Discussions

**Figure 1:**
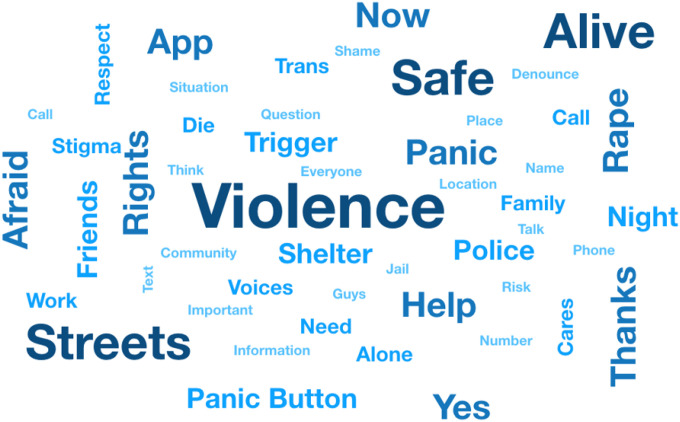
Word cloud of the 50 most frequent words mentioned in Focus Groups Discussion.

The descriptive analysis of qualitative data included plotting a ‘word cloud’ to represent word frequency visually. The more commonly the term appeared within FGDs under analysis, the larger the word appears in Figure 1. A brief visual analysis highlights the clear impact of violence within this community. The most mentioned word was “violence”, followed by “safe,” “streets,” and “alive.’ Many participants are street sex workers, and the perception is frequent that “*We go out with a client, but we don’t know if we will come back alive*”. Participants also expressed their gratitude for having their voices and concerns heard and being included in developing and improving the Rainbow Resistance - Dandarah app.

### App Acceptability

Acceptability was defined as the willingness of SGM persons to use the Rainbow Resistance- Dandarah app. Participants said the community was willing and interested in utilizing the app. Many FGDs participants mentioned the importance of the collaborative strategy utilized in the study, where the SGM community was consulted to identify their specific needs, was invited to compose a highly active Community Advisory Board, tested the app prototype, and had the opportunity to criticize and contribute to improving the app.

“We know the way the LGBT community is extremely technological, like these things, will use it here, it’s a lot, and it stays at the national level, it [will be] a lot of denunciation, a lot of denunciation… We need this”

“Yesterday, the test was with trans people. And then, we did different things because the needs and worries of the trans population are completely different from the LGB population. And this participation of the ENTIRE LGBT community is just… Amazing. I mean, this makes all the difference. That’s not a study that some white, rich researcher planned and decided to do. Do you know what I mean? That’s something built for and by our community. And OMG, I’m so proud to be here! We’re making history!”

### App Anticipated usability

Anticipated usability was defined as the extent to which SGM persons found the program useful to report violent episodes and access support. Many participants identified problems to be further addressed, mostly related to the mapping feature. Standardized maps like Google Maps and Waze frequently miss out on specific locations, such as informal settlements. Favelas (slums or shantytowns) are located within or on the outskirts of the country’s large cities, such as Rio de Janeiro. Many of Favela’s streets are not included in formal maps of those cities. Therefore, when utilizing the ‘panic button’, several participants reported that their location was unavailable in the app.

“The first and most important mistake is the location. Like, when I needed to use the panic button, the app didn’t find the street where I was. And we live in favelas, right? So, you guys gotta update this. There gotta be some sort of check or maybe another feature where we could enter our correct location…”

Another usability problem was related to the questionnaire that needed to be filled out before pressing the panic button:

- *Now, do you see here? I’ll try to press the panic button… Now gals, let’s pretend this guy here is attacking me*…
- *Okay* [laughs]
- *And check it out, I gotta fill all that stuff before pressing the panic button*… *I mean, it’s impossible right?*
- *Yeap, it’s like ‘Hey yow, I know you wanna attack me, but chill man*… *Wait just a sec until I fill all that stuff and then you attack me, okay?*
- *She is dead already*… [laughs]
- *I mean*, *this gotta be quickly*, *right?”*

### App Feasibility

Feasibility was defined as the extent to which the program could be made readily available and implemented among the SGM from Brazil. The vast majority of participants perceived the app as a huge success and understood that it would be available soon after the pilot tests, and features review was finished.

- *“ Y’all, this app is like… It’s fierce! Like, they got together Fiocruz, ANTRA* [Brazilian National Transgender Association], *and ABGLT* [Brazilian National LGBTQ+ Association]. That’s *dope!*
- *So, we want to thank you for the opportunity for us*… *I speak on behalf of everyone here so that we can speak out, understand?*
- *I’m emotional here…”* [cries]

The revised app was launched in December 2019 and became a formal strategy from the Brazilian Ministry of Health through an active partnership with the Oswaldo Cruz Foundation, its research branch.

### Additional feedbacks

Feedback on the app’s look, feel, and content was also gathered to enhance acceptability. Participants requested the inclusion of additional information such as legislation, addresses of local services, non-governmental organizations, shelters etc. A few participants also suggested additional features highlighting success stories of well-known SGM activists and not-so-well-known members of the community as well. Those features are under development.

### App Initial Impact

Following the pilot testing, our team reviewed all suggestions and improved/revised a few app features. Between December 2019 and August 2022, over 4,000 SGM persons have been using the app to report violence, crowdsource information about safe places for SGM persons, and connect with other users to offer support for violence survivors in need.

Unfortunately, during the COVID-19 pandemic, the levels of violence against SGM persons from Brazil were even higher: around 1 SGM person was murdered in the country every day between 2020–2021. Levels of physical and sexual violence were also rampant but continued to be under-reported to local authorities. The Rainbow Resistance - Dandarah App helped to connect survivors with a broad range of services, including shelters, post-rape care, and pro bono legal, psychological, and social support. App users reported a deep sense of gratitude for having the app downloaded. Many even mentioned that it might have saved their lives, as we can see from the quotes below:

“Doc, let me tell you… It was late at night, and I was waiting at the bus stop. Then a car approached me super slowly, and when it was in front of me, it stopped. I got the chills, you know? Four white men got out of the car, and I knew they wanted to beat me just by the look in their eyes… They started laughing and teasing me. One of them opened his pants and asked the others to hold me… I knew I was gonna be raped, and just like many Black trans women before me, I might end up dead in an alley… I quickly opened the app and touched the panic button as my last breath of hope. No more than 5 minutes later, three trans women showed up, screaming and telling them the cops would be there any minute. They are sex workers and happened to be on the other street, less than five minutes from me!! You know that when you press the panic button, it shares your location with friends, right? So, they knew I was in trouble. Those guys, I could see they were freaking out! They ran to the car and left me alone with my friends, no harm done. I was shaking and scared but safe. My friends took me home. I reported the incident on the app but didn’t go to the police… Like, they would laugh in my face, like always… I never used that bus stop again… [get emotional] Honestly, your app saved my life that night…”

(Black Transgender woman from Rio de Janeiro, 24yr)

“Like, I was living with my partner when the pandemic got really bad… He was a truck driver, and I was used to staying all by myself at home. He was always on the road, you know? But when COVID came, he was laid off… So, I was supporting him and the house… Like, I’m a sex worker, and he could only eat if I got laid with other guys, some of whom he knew… And changed… Like, he started drinking every day and got really violent with me. I don’t make a lot of money, so I had to save for the bills and groceries, right? But he wanted money every day to get drunk. One day I stood tall and said, “No! That’s enough! We will starve to death like this.” Girl, he got possessed! I never saw such a scary face in my life… And he punched me in the face, took my purse with all the money I got from the night before, and started kicking me really hard… I thought I was gonna die that day… But I had my cell phone in my pocket, and I don’t know how, but I managed to press the panic button when he was not looking. Guess what? A few minutes after, a client of mine, who is in the army, showed up at my door!!! He was arrested, and the social worker took me to a safe shelter. I mean, if it was not for your app and this panic button, I could be dead!”

(Indigenous Transgender women from Manaus, Amazon capital, 26yr)

“When things got bad, with COVID, you know? My life became a mess! I had no school, and all my classes were online… And my dad hates me. He really does. Like, he doesn’t accept a lesbian daughter, you know? And he was also working from home, so I was like… Trapped. He looked at me with hate in his eyes. I could tell… And my mom works cleaning homes all day long… I used to lock my door ‘cause I was really afraid… But one day, I forgot to lock my bedroom door and napped after my morning class… When I woke up, my dad was staring at me, and he said: ‘Now you will learn to like a man!’ I knew exactly what he meant… And he started taking off my clothes and put my hand inside his pants… It was sooo gross, OMG…, but I had my phone under my pillow, you know? And I pressed the RISE panic button, hoping my mom or someone else would see it… He beat me badly, and I don’t remember much after that… But when I woke up, my mom was home with a woman police officer, and they were taking my dad to jail… Then they told me that my dad raped me… [gets emotional] I’m still shaking just by telling you this… When they arrived, my dad had a gun and almost killed me, but I was unconscious… Thankfully the police arrived quickly and got him before that… My dad is still in jail and won’t leave any time soon, I’m seeing a psychologist that mom found on your app for free, and she is helping me… I’m still really depressed, you know? But I’m glad to be alive and free to be who I am. And my mom is also free from him… Thanks to this app, I’m still alive, and my dad is in jail…”

(White lesbian from Sao Paulo, 17yr)

## Discussion

SGM persons frequently experience hate crimes, violence, attempts to change their sexual orientation forcibly and/or gender identity, and discrimination in education and employment settings, healthcare facilities and public spaces (Tremblay et al., 2018; Mallory et al., 2015). This population also faces high rates of profiling, discrimination, and harassment by law enforcement officers, influencing their willingness to report crimes (Mallory et al., 2015). Despite this scenario, relatively few strategies have been made to systematically address the effects of discrimination and violence against SGM persons.

The current study presented the development and first feedback about the impact of a mHealth intervention for SGM persons from Brazil: The Rainbow Resistance - Dandarah. The strategy was developed to increase reports of discrimination and violent episodes and to provide support for SGM surviving those experiences in one of the most dangerous countries in the world for SGM persons: Brazil.

According to participants, this mHealth strategy was perceived as an important, effective, and accessible strategy for SGM surviving violence. Other studies have implemented mHealth interventions to improve SGM’s mental health and quality of life (Leluțiu-Weinberger et al., 2018; Fleming et al., 2017; Dubov et al., 2021). However, the vast majority of mHealth strategies available for persons experiencing discrimination and violence were developed, having as key users cis, heterosexual persons (Draughon Moret et al., 2022). To the best of our knowledge, the Rainbow Resistance - Dandarah is the first mHealth intervention developed so far for SGM to survive discrimination and violence in Latin America.

According to initial results, the Panic Button is a highly important feature for SGM persons who are experiencing an assault. By pushing the red Panic Button, the app sends their current position and address (in the form of a Google Maps link) to five numbers registered in their panic contact list. Such a list can be a true lifesaver, as mentioned by users’ feedback. There are many apps specifically developed for personal safety and violence prevention. However, the vast majority was not developed to address the specific needs of SGM persons (Ford et al., 2022)

SGM persons usually have higher rates of mental health issues than people in the general population (Moagi et al., 2021). SGM survivors of violence have even higher rates of PTDS, generalized anxiety, depression, and suicidality (Miller et al., 2017; Henry et al., 2021). Therefore, ongoing improvements of the Rainbow Resistance: Dandarah App will include a prompt for all users reporting a violent episode to conduct mental health screening. Those with higher scores will be referred to the nearby mental health service for further assessments and treatment.

Through crowdsourcing, app users now have access to an updated feature that allows them to identify SGM-welcoming and safe services. This additional feature is key to improving SGM persons’ access to healthcare services. Several studies have identified that SGM persons experience additional barriers to accessing healthcare when compared to the general population (Hafeez et al., 2017; Akré et al., 2021; Ercan Sahin et al., 2020). By identifying SGM-welcoming and welcoming services, the research team in partnership with the Brazilian Ministry of Health will be able to develop tailored training for services perceived as unwelcoming.

Brazil is a country where at least one SGM person is murdered every day, and discrimination/violent episodes are highly underreported. In this context, the Rainbow Resistance: Dandarah App is not only collecting pivotal and inexistent data about violence and discrimination of SGM persons but also connecting those in need with a broad range of supportive services.

### Strengths and Limitations

The current study included a large but convenient sample of SGM persons actively utilizing the *Rainbow Resistance: Dandarah App*. Future work is needed to evaluate the impact of app use over time, for instance, improving crime reporting to the police and/or improving access to healthcare.

Within the broad SGM community, transgender and gender non-conforming persons and radicalized SGM persons experience a higher prevalence of discrimination and violence. Therefore, it is pivotal to consider the unique needs of this population and perhaps include additional features that might help to address barriers to receiving healthcare among these underserved populations.

The SGM community experiences additional barriers to reporting experiences of discrimination and violence and accessing a broad range of services needed after an assault (e.g., post-rape care, shelters, psychological counselling, legal and social support etc.). Therefore, innovative approaches to improve the reporting of discrimination/violence, like the Rainbow Resistance: Dandarah App, are urgently needed for the SGM community. The presented results are descriptive and preliminary. Ongoing and longitudinal analyses are still needed and will be conducted over time.

The usability evaluation was conducted during FGD, with a moderator always present. The group setting and the presence of researchers may have inhibited participants’ responses. Therefore, assessing Rainbow Resistance: Dandarah App usability may be useful when there is no researcher present through self-report as well. Additional evaluations should also include previously validated scales and a more robust design to identify the App efficacy.

Despite those limitations, it is important to highlight that the intervention was conducted in one of the most dangerous countries for SGM to live in. Brazil records at least one murder of an SGM person every day, interventions are scarce, and violence/discrimination reporting is almost absent due to police SGM phobia and victim blaming. To the best of our knowledge, this is the first nationwide intervention implemented in Brazil that allows SGM persons to report violence/discrimination and access a broad range of supportive services. Additionally, the Dandarah: Rainbow Resistance App includes crowdsourcing and mapping strategies, allowing users to identify services that are more welcoming and accessible for SGM persons. Those additional features might be highly important to decrease barriers to accessing services among this highly vulnerable population.

## Conclusion

The Rainbow Resistance: Dandarah App is the result of a broad and collaborative work that included the Brazilian SGM community, healthcare professionals, researchers, and government representatives. The results of this pilot study indicate that Rainbow Resistance: Dandarah App is an engaging, acceptable, and potentially effective intervention. Participants reported many advantages of using the app, such as reporting violence, connecting with peers, and accessing help in real-time. This study provides initial support for the acceptability and usability of Rainbow Resistance as a mHealth intervention designed by and for the SGM community from Brazil. The next steps involve further app refinement, including mental health screening, referrals, and more substantial feasibility testing. It is also essential to evaluate if app users can connect with healthcare services, stratifying by type of discrimination/violence experienced and by sub-population.

## Figures and Tables

**Table 1.** Selected socio-demographic characteristics of SGM persons utilizing the Rainbow Resistance - Dandarah app, 2019–2022

Sociodemographic characteristics (n=4,114)	Frequency (%)
**Gender Identity**	
Cisgender Men	2095 (50.9)
Transgender Men	209 (5.1)
Cisgender Women	788 (19.2)
Transgender Women	238 (5.8)
Travesties	75 (1.8)
Other	477 (11.6)
NA	232 (5.6)
**Gender Identity “Other” (n=360)**	
Non identified	163 (45.3)
Family/allies	20 (5.6)
Intersex	58 (16.1)
Nonbinary	115 (31.9)
Queer	4 (1.1)
**Sexual Orientation**	
Bisexual	599 (14.6)
Gay	1788 (43.5)
Heterosexual	412 (10.0)
Homosexual	391 (9.5)
Lesbian	527 (12.8)
Pansexual	298 (7.2)
Other	99 (2.4)
**Sexual Orientation “Other” (n=61)**	
Androsexual	2 (3.0)
Asexual	31 (47.0)
Non identified	14 (21.2)
Family/allies	1 (1.5)
Gynesexual	1 (1.5)
Other sexual expressions	11 (16.7)
Sexual orientation towards more than one gender	6 (9.1)
**Intersex**	
Yes	280 (6.8)
**Race/Ethnicity**	
Asian	73 (1.8)
White	1948 (47.3)
Indigenous	35 (0.9)
Mixed	1265 (30.7)
Black	670 (16.3)
Other	17 (0.4)
NA	106 (2.6)
**Age (years)**	
10–19	807 (19.6)
20–29	1,699 (41.3)
30–39	861 (20.9)
40–49	434 (10.5)
50–59	105 (2.6)
≧60	20 (0.5)
NA	188 (4.6)
**Disability**	
Yes	93 (2.3)

## Data Availability

The data included in this study are stored within the Brazilian Ministry of Health at the National School of Public Health from Oswaldo Cruz Foundation - ENSP/FIOCRUZ (https://ensp.fiocruz.br). To access similar data, it is necessary to apply for access with Dr. Angelica Baptista da Silva from ENSP/FIOCRUZ.

## Abbreviations

ABGLT: Brazilian National LGBTQ+ Association
ANTRA: Brazilian National Transgender Association
CAB: Community Advisory Board
CBPR: Community-Based Participatory Research
FGDs: Focus Groups Discussions
IDIs: In-Depth Interviews
SGM: Sexual and Gender Minorities
